# The Impact of Migraine on Posterior Ocular Structures

**DOI:** 10.1155/2015/868967

**Published:** 2015-02-12

**Authors:** Süleyman Demircan, Mustafa Ataş, Sevgi Arık Yüksel, Melek D. Ulusoy, İsa Yuvacı, Hasan Basri Arifoğlu, Burhan Başkan, Gökmen Zararsız

**Affiliations:** ^1^Kayseri Training and Research Hospital Eye Clinic, 38010 Kayseri, Turkey; ^2^Kayseri Training and Research Hospital Neurology Clinic, 38010 Kayseri, Turkey; ^3^Biostatistics Department, Erciyes University, 38039 Kayseri, Turkey

## Abstract

*Purpose*. To investigate the thickness of the retinal nerve fiber layer (RNFL) and choroid in patients who have migraines in comparison to healthy controls.* Methods*. This study included 76 eyes and patients in the migraine group, 36 with aura (MWA group) and 40 without (MWoA group), and 38 eyes as control subjects. The RNFL and macular thicknesses were analysed with standard OCT protocol while choroidal thickness was analysed with EDI protocol in all subjects. Choroidal thickness was measured at the fovea, 1500 *µ*m nasal and 1500 *µ*m temporal to the fovea in a horizontal section.* Results*. The mean RNFL thickness for nasal and nasal inferior sectors was significantly thinner (*P* ≤ 0.018) in the migraineurs' eyes than in those of the controls, as was the mean choroid thickness at the fovea and measured points (*P* < 0.0001). However, the mean macular thickness was not significantly different between the groups.* Conclusions*. This study suggests that migraine leads to a reduction in the peripapillary RNFL thickness and to thinning in choroidal structures. These findings can be explained by a chronic ischemic insult related to migraine pathogenic mechanisms and these findings are considered supportive of the relationship between glaucoma and migraine.

## 1. Introduction

Migraine is a common episodic neurovascular disorder characterized by recurrent attacks of typically throbbing, unilateral, and often severe headache with associated features such as nausea, vomiting, and hypersensitivity to light, sound, and smell. In one-third of migraines, the headache is preceded by transient neurological symptoms (visual, motor, or somatosensory) lasting for up to an hour or sometimes for several days and known as an aura [1, 2]. Migraine is clinically divided into two main subtypes based on the absence or presence of an aura: migraine without aura (MWoA) and migraine with aura (MWA) [2]. Migraine affects approximately 15% of the general population, making it the third most frequent disease worldwide in both genders [3]. The neurovascular system is one of the most important mechanisms involved in the pathogenesis of migraine. Migraine episodically leads to the activation and sensitization of the trigeminovascular system (TGVS). This system is thought to play an integral role in regulating vascular tone and in the transmission of pain signals. The TGVS consists of the trigeminal nerve and nerve fibers that innervate the network of extra- and intracranial meningeal blood vessels and the brain stem. In detail, the peripheral terminations of the TGVS are also located in extracranial soft tissues such as structures in the eyes and arteries, as well as in intracranial structures. Activation of the TGVS stimulates the release of vasoactive neurotransmitters from peripheral terminations of the trigeminal nerve which cause the vascular and inflammatory changes associated with migraine pain. It is possible that not only the hypoperfusion in migraineurs but also a hyperperfusion, which may even follow each other, may involve areas other than the brain, including the retina and choroid [4–7]. Although the vasoconstriction of cerebral and retinal blood vessels is a transient phenomenon, the chronic nature of migraines might cause permanent structural abnormalities in the brain as well as in the retina and choroid. Structurally and functionally normal retinal and choroidal vasculature is essential for the function of the retina, as abnormal choroidal blood volume and/or compromised flow can result in photoreceptor dysfunction and death [8]. Changes in the retinal and choroidal blood flow may lead to an alteration of the retinal and choroidal vasculature in migraine. Despite this, few studies have evaluated whether the retina and choroid are involved in migraine patients.

The aim of this study was to compare the thickness of the retinal nerve fiber layer (RNFL), macula, and choroid in the eyes of migraine patients with age-matched healthy subjects using enhanced depth imaging (EDI) optical coherence tomography (OCT).

## 2. Methods

### 2.1. Study Population and Design

This prospective, observational, cross-sectional study was conducted at the eye and neurology clinics at the Kayseri Training and Research Hospital on 76 patients who had previously been diagnosed as having migraine, with or without aura, according to the criteria of the Headache International Society [2]. The control group consisted of 38 age- and sex-matched healthy controls. Control subjects were required to have had fewer than three headaches in the past year and to have never experienced a migraine. The study protocol was approved by our institutional review board and performed according to the Declaration of Helsinki. Written informed consent for participation in the study was obtained from each patient.

All selected eyes had a best-corrected visual acuity (BCVA) of 0.20 or better (logarithm of the minimum angle resolution (logMAR)). A normal optic disc was defined as having a cup/disc area ratio less than or equal to 0.4 and a neuroretinal rim with no glaucomatous changes such as localized rim loss, thinning of the rim, or peripapillary hemorrhages seen ophthalmoscopically. If both eyes met all the inclusion criteria, only one eye per patient was selected based on the laterality of migraine. The exclusion criteria were pregnancy, smoking, organic eye disease, previous ocular trauma, intraocular surgery, cataract, glaucoma, strabismus, eccentric fixation, laser treatment, neurological disorders, retinal or optic nerve disorders, and diabetic or hypertensive retinopathy.

### 2.2. Examination Protocol and Study Measurements

Each participant underwent a comprehensive neurologic and ophthalmologic examination, which included the following tests: BCVA (logMAR, Early Treatment Diabetic Retinopathy Study (ETDRS) chart), slit-lamp biomicroscopy, gonioscopy, dilated funduscopic examination with a 90-diopter lens, and intraocular pressure (IOP) (Goldmann applanation tonometry), axial length (AL), and OCT measurements. The IOL Master (Carl Zeiss Meditec, Dublin, CA) was used for the ocular biometry to measure the corneal curvature and AL, repeating the measurements until five valid values were obtained. The AL was measured from the corneal vertex to the retinal pigment epithelium. The AL measurements were interpreted based on the signal-to-noise ratio (SNR) above 2.0 and the appearance of the graphs. EDI-OCT software (version 5.6.4.0; Spectralis OCT Heidelberg Engineering, Dossenheim, Germany) was used to assess the thickness of the RNFL, macula, and choroid.

### 2.3. OCT Imaging

The Spectralis OCT device was used to assess the peripapillary RNFL thickness and the macular thickness. Scans for all participants were performed with pupillary dilatation under the same intensity of dim room lighting by the same experienced technician. An internal fixation target was also used in all scans with the real-time eye tracking system to adjust for eye motion. The macular thickness (*μ*m) was determined automatically and analyzed by the OCT software. The fast macular thickness map included a 25-line raster volume scan, 20 × 20 degrees, and was centered on the fovea. In the raster scans utilized for the macular measurements, the scans were obtained in high-speed mode with the automated real-time (ART) feature enabled and set at 9 frames. The infrared scanning laser ophthalmoscope scan angle was set at 30 degrees for all acquired scans. We selected the macular map analysis protocol on the Spectralis to display the numeric averages of the measurements for each of the nine subfields as defined by the ETDRS circle grid. The diameters of the concentric circles were 1, 3, and 6 mm for the macular scan. The results obtained from the macular scan were classified by segments.

The peripapillary RNFL thickness parameters were automatically calculated by the fast-RNFL mode. Scans were obtained in high-speed mode with the ART feature enabled and set at 16 frames. This software provided a thickness profile across the temporal-superior-nasal-inferior temporal areas of the standard 12-degree circular scan. The software also calculated average thickness values (*μ*m) globally and for each of the 6 sectors centered on the optic disc (temporal, temporal superior, temporal inferior, nasal, nasal inferior, and nasal superior). Spectralis uses a signal-to-noise estimate (SNR in dB) for the quality score. After all exposures, the scans not centered or with SNR < 20 dB were excluded from the study.

The choroidal thickness was measured by EDI using the spectral-domain OCT. Each section, consisting of 30 average scans, was obtained in a 15 × 30-degree rectangle centered at the macula. Choroidal thickness was determined as the distance from the outer surface of the hyperreflective line, referred to as the “retinal pigment epithelium” layer, to the hyperreflective line of the inner scleral border. Choroidal thickness was measured at the fovea, 1500 *μ*m nasal and 1500 *μ*m temporal to the fovea in a horizontal scan section.

**Figure 1:**
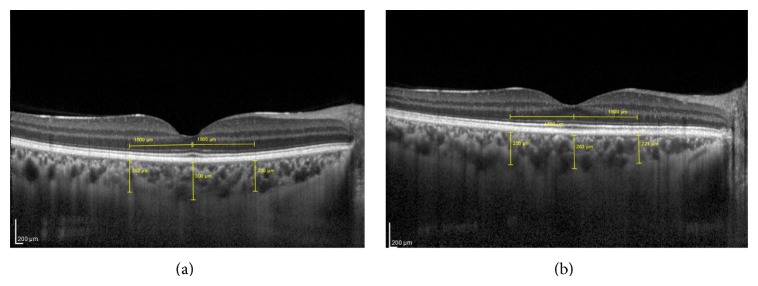
(a) shows picture of the scanning imaging obtained from control's choroid thickness. (b) shows picture of the scanning imaging obtained from migraineur's choroid thickness.

Figures 1(a) and 1(b) show pictures of the scanning imaging obtained from two of our subjects. The axial resolution is 3.9 mm digital, which is the same as for routine OCT images obtained by Spectralis OCT. Measurements were evaluated by two independent ophthalmologists (Süleyman Demircan and Mustafa Ataş), and the mean value was generated for analysis. The measurements were performed at the same time of day to avoid diurnal fluctuations.

### 2.4. Statistical Analysis

Normality assumptions were assessed using MVN (http://www.biosoft.hacettepe.edu.tr/MVN/) software. Chi-square analysis was used to compare the differences for categorical variables. Kruskal-Wallis *H* test and one-way analysis of variance (ANOVA) were used to compare the differences for continuous variables. Tukey's and Tamhane's *T* ^2^ tests were used for multiple comparisons. One-way multivariate analysis of variance (MANOVA) was used for multivariate analysis. Analysis was conducted using R 3.1.0 (http://www.r-project.org). *P* values less than 5% were considered to be statistically significant.

## 3. Results

This study included 76 eyes in the same number of patients in the migraine group, 36 in patients with aura and 40 in patients without aura, and 38 eyes in 38 participants in the control group. The mean ages were 38.8 ± 11.6, 37.6 ± 15.7, and 37.9 ± 11.1 years in the MWA, MWoA, and control groups, respectively. The mean attack frequency was 3.1 ± 1.6 attacks/month in the MWA group and 2.9 ± 1.6 attacks/month in the MWoA group, respectively, and the mean duration of disease was 10.8 ± 8.1 years and 11.1 ± 7.9 years. There was no significant difference between the mean duration of disease (*P* = 0.928) or the mean number of attacks (*P* = 0.688). There were also no statistically significant differences between the migraineurs and the healthy subjects in terms of sex, BCVA, AL, or IOP (Table 1). The average RNFL thicknesses (*μ*m) for nasal and nasal-inferior sectors were significantly less (*P* ≤ 0.018) in the eyes of the migraine group than in those of the control group (Table 2). The MANOVA did not reveal any significant difference between the MWoA, MWA, and control groups (*P* = 0.139). The mean duration of migraine or the mean number of attacks of the migraine patients was not statistically significantly correlated with reduction in the average RNFL thicknesses (*μ*m) for nasal and nasal-inferior sectors (*P* > 0.05). Similarly, the mean choroid thickness (*μ*m) at the fovea, 1500 *μ*m nasal and 1500 *μ*m temporal to the fovea, was significantly less (*P* < 0.0001) in the eyes of the migraine group than in those of the control group (Table 3). However, there were no significant differences between the two groups in the mean macular thickness (*μ*m) in any sector (Table 4).

## 4. Discussion

Migraine is a neurovascular disorder with complex and poorly understood underlying mechanisms. Most current models of migraine pathogenesis claim that a condition of brain hyperresponsivity to several exogenous and endogenous stimuli may underlie the susceptibility to migraine attacks. However, the exact pathophysiological mechanisms leading to the onset of an attack remain under debate. Cortical spreading depression (CSD) is the underlying mechanism of the migraine aura characterized by brief neuronal excitation, which is followed by a prolonged inhibition of neuronal activity [9]. It may also trigger the headache phase of migraine attacks by activating and sensitizing the trigeminovascular system (TGVS), initiating a series of neural, vascular, and inflammatory events that result in pain [9, 10]. The TGVS consists of the trigeminal nerve and nerve fibers that innervate the network of extra- and intracranial meningeal blood vessels and the brain stem. This system is thought to play an integral role in regulating vascular tone and in the transmission of pain signals. In detail, the peripheral terminations of the TGVS are located in extracranial soft tissues such as muscles, eyes, ears, skin, subcutaneous tissue, nasal cavities, arteries, and periosteum, as well as in intracranial structures or venous sinuses and the vagus and glossopharyngeal nerves. Activation of the TGVS stimulates the release of neuropeptides from peripheral endings of the trigeminal nerve which cause the vascular and inflammatory changes associated with migraine pain. It is believed that these neuropeptides play a role in causing a sterile neurogenically driven inflammation of the meningeal blood vessels wall (the dura mater), including mast cell degranulation and changes in postcapillary venules including extravasation of plasma proteins and platelet aggregation, and in maintaining the migraine pain. Neurogenic plasma extravasation in both dura and the retina can be seen after electrical stimulation of the trigeminal ganglion in experimental animals [11, 12]. This presumed neurogenic inflammation can also occur in the choroid, and Dadaci et al. [13] and Karalezli et al. [14] reported that it may result in an increased choroidal thickness during a migraine attack.

Studies have detected an increased risk for ischemic stroke in patients who had migraine with aura [15–18]. Vasospasm emerging prior to or during the pain has been considered to occur concurrently in tissues located outside the brain, and, by extension, local infarctions have been considered to lead to histopathological and functional disorders at the tissue level. Killer et al. [6] demonstrated a reduction in blood flow in the lower temporal artery in a patient who had a visual area defect in the left eye during a migraine attack. Using colored Doppler ultrasonography, Kara et al. [19] found a reduction in blood flow at the level of the central retinal and posterior ciliary arteries in migraine patients without aura relative to healthy individuals during intercritical migraine periods.

Using scanning laser polarimetry in studies in migraine patients both with and without aura, Tan et al. [20] found that the RNFL thickness was unaffected. In contrast, Martinez et al. [21] reported that the mean RNFL average thickness parameter was significantly less in the migraine subjects than that in the control group, although there were no differences between the migraine subjects and controls in the mean RNFL thickness in the superior and inferior areas. In another study, Martinez et al. [22] found that the mean RNFL thickness in migraine patients was similar to that of healthy individuals, with only the thickness of the temporal quadrant. The RNFL thickness parameters correlate with the migraine disability assessment score (MİDAS), number of attacks, and length of migraine history.

Another interesting point of the Martinez et al. [22] study is that the length of migraine history critically influenced the RNFL thickness. The average RNFL thicknesses for global and superior, inferior, and temporal quadrants were significantly thinner in the eyes of the length of migraine history at or above 15 years than in those of the length of migraine history below 15 years. This finding raises questions regarding whether migraine could be associated with glaucoma, although this is controversial. The functional vasospasm of brain and retrobulbar vessels is linked to the pathogenesis of migraine. Attacks of migraine may be related to decreased blood flow in the retina and optic nerve. This in turn may lead to unstable ocular perfusion and thereby to ischemia and reperfusion damage in duration of migraine history. Ocular blood flow changes are involved in both the pathogenesis of glaucoma and the progression of glaucomatous damage. Drance et al. [23] reported that normal tension glaucoma patients with migraine had a faster course of visual-field deterioration than those without a history of migraine. In addition, a statistically significant correlation was found between the migraine severity and RNFL thickness.

Gipponi et al. [24] found significant thinning in the RNFL thickness in the upper quadrant but found no differences in the foveal thickness and macular volume in female migraine patients relative to healthy women. We found that the mean duration of migraine or the mean number of attacks of the migraine patients was not statistically significantly correlated with the reduction in the average RNFL thicknesses (*μ*m) for nasal and nasal-inferior sectors (*P* > 0.05). Similar to our results, Gipponi et al. [24] found no correlation between reduction in RNFL thickness and duration and frequency of migraine. They proposed that the reduction in RNFL thickness strictly related to the presence of migraine. Yülek et al. [25] found no statistically significant differences between the control group and the migraine patients for the retinal thickness in any of the quadrants (*P* > 0.05). In this study, the average RNFL thickness was significantly thinner in the migraineurs than in the control subjects, and the ANOVA did not reveal any significant difference between the MWoA, MWA, and control groups. Our results are different from their results. We found that the average RNFL thicknesses (*μ*m) for nasal and nasal-inferior sectors were significantly less (*P* ≤ 0.018) in the eyes of the migraine group than in those of the control group. However, the average global RNFL thickness was no statistically significant difference between the control group and the migraine patients. The MANOVA did not reveal any significant difference between the MWoA, MWA, and control groups (*P* = 0.139).

Evidences from the literature suggest that there is a stronger vascular risk associated with MWA than with MWoA which focuses attention on the importance of the aura in vascular disease [15–18]. We speculated that a putative vascular mechanism linking migraine and modification of posterior ocular structures is specifically referred to MWA. However, we did not find any differences between RNFL, macula, and choroid thicknesses in MWA and MWoA subgroups. This result may be explained by the fact that there is a common putative pathogenic mechanism for both migraine types. Our findings suggest that some patients, not only MWA but also MWoA, may be at increased risk for possible migraine-related ischemic events. It is likely that there may be a correlation between migraine-related structural brain changes and ocular changes. The structural brain studies indicated that female migraineurs, both with and without aura, are at increased risk of deep white matter and brainstem hyperintensities. These hyperintensities and posterior circulation territory infarct-like lesions are believed to be of ischemic origin [26]. In a recent study Palm-Meinders et al. [27] evaluated the pathogenesis and relevance of migraine-related structural brain changes and their possible relation with ischemic events. Women in the migraine group had a higher prevalence and a greater increase of deep white matter hyperintensities than women in the control group. Although migraine was associated with a higher prevalence of infratentorial hyperintensities at follow-up, there were no significant associations of migraine with progression of infratentorial hyperintensities or posterior circulation territory infarct-like lesions among women. In addition, the number of migraines, frequency of migraines, migraine severity, type of migraine (MWA or MWoA), and migraine therapy were not associated with lesion progression [27]. Similarly, we found no correlation between the mean duration of migraine, the mean number of attacks of the migraine, and type of migraine and reduction in RNFL thickness.

A limitation of our study is that most of the treatments used for migraine attack are vasoactive (i.e., triptans). Although triptans are generally considered safe for use in migraine, Schmetterer et al. [28] found that ocular fundus pulsations were slightly reduced after sumatriptan. Measurement of ocular fundus pulsations indicates that sumatriptan also has a small vasoconstrictor action on resistance vessels. At least theoretically, the migraine-ischemic insult link may be the consequence of the unfavourable effect of migraine-specific drugs (i.e., triptans or ergot alkaloids). Therefore it may be speculated that their vasoactive properties are a confounding factor for our results of the study.

An additional study limitation is that severity of migraine or MIDAS disability score was not evaluated. Martinez et al. [22] showed that the average RNFL thickness was significantly correlated with MIDAS. However, Yülek et al. [25] found that the visual analogue scale score of the migraine patients was not statistically significantly correlated with any of the parameters, while the length of migraine history was negatively correlated with the average RNFL thickness, supporting a possible association between these pathologies. In another study, Ekinci et al. [29] found that the thinning of the RNFL and ganglion cell layer was detected only in the MWA group when groups were analysed. However, Kirbas et al. [30] used SD-OCT to investigate the RNFL thickness and macular changes in patients with chronic migraine (CM) without visual impairment in comparison to healthy controls. The average RNFL thickness was not significantly thinner in the patients with CM, but the mean superior quadrant RNFL thickness was significantly less in the CM patients than in healthy controls. We found that the mean duration of migraine or the mean number of attacks of the migraine patients was not statistically significantly correlated with reduction in the average RNFL thicknesses (*μ*m) for nasal and nasal-inferior sectors (*P* > 0.05). Similar to our findings, migraine duration and the frequency of attacks were unaffected by the RNFL thickness, and the macular changes were not significantly different between the CM patients and healthy controls. Similar to our results, Sorkhabi et al. [31] found that RNFL thickness was significantly thinner only in the nasal quadrant in migraineurs relative to a control group, and there was no statistical difference between the two migraine subgroups. In addition, the reduction in the average RNFL thicknesses for nasal quadrant was not significantly correlated with the mean duration of migraine from diagnosis, the mean number, or the severity of attacks of the migraine patients.

The discrepancy between the results of these studies may be explained by the difference in methods used and the migraine severity [20–22]. The mean age of the participants in the studies by Martinez et al. [21, 22] was less than that in the study by Tan et al. [20]. They also used scanning laser polarimetry or TD-OCT technology. However, the improved resolution of SD-OCT can detect fine structural changes. In addition they did not take the AL into consideration. AL could influence the measured thickness of the retinal structures due to the reflectance directionality, which can degrade the accuracy of technologies assessing RNFL thickness.

The choroid is responsible for most of the ocular blood flow, which is the highest of any tissue in the body, to supply the normal metabolic demands of the outer retina [32]. Migraine is known to reduce blood flow at the level of the central retinal and posterior ciliary arteries; a thinning of the choroid layer is an expected clinical outcome in migraine patients [29]. However, Dadaci et al. [13] and Karalezli et al. [14] noted that an increased choroidal thickness during the migraine attack period could reflect an alteration of ocular circulation. However, the other studies reported a reduction in choroidal thickness during the migraine attack period in MWA and MWoA groups [33, 34]. The discrepancy between the results of these studies may be explained by the fact that both groups of migraine evolve from hypoperfusion to hyperperfusion during their time course, although perhaps with a difference in intensity. Like Ekinci et al. [29], we found that the choroidal thickness was significantly decreased in migraine patients, with or without aura, in comparison to healthy participants. This can be explained by the detrimental effect of migraine on the vascular system in chronic nature of the disease.

A reduction in the RNFL thickness has been reported in migraine patients and is most likely a consequence of retinal ischemia. In contrast to the choroidal vessels, which have an intense autonomic innervation, the intraocular portion of the retinal vessels has no autonomic innervation. The superficial layer of the optic nerve head is supplied by small branches originating from the central retinal artery, while the anterior part of the optic nerve is supplied primarily by the short posterior ciliary arteries and choroidal vessels. An alteration in the quality of the blood supply in the optic nerve or RNFL could be indirectly triggered to a greater or lesser extent by altered rheological characteristics of the blood, local vasospasm, or changes in the physiological and/or physical characteristics of the blood vessels implicated in the process. It could be hypothesized that an alteration in the quality of blood supply in the anterior optic nerve head leads to an oligemic-hypoxic insult, which then contributes to ganglion cell death. Thus, alterations in these structures may be associated with diseases of the optic nerve. In fact, decreased blood flow in the ophthalmic and posterior ciliary arteries has been shown to be associated with the progression of glaucoma [35]. The chronic ischemic insult related to migraine pathogenic mechanisms may cause structural changes in the lamina cribrosa which have been implicated in the pathogenesis of glaucomatous optic neuropathy [36]. The laminar region of the optic nerve head is the principal site of the retinal ganglion cell axonal insult in glaucoma. According to our OCT findings, Seong et al. [37] found that patients with normal tension glaucoma had reduction in superior and inferior quadrants, similar to our patients with MWA or MWoA. Further studies are needed to determine the alterations in the lamina cribrosa in patients with migraine. In addition, alterations in the ocular and systemic circulation have been demonstrated in patients with glaucoma [38].

The OCT results in this study suggest that the migraine pathological process could have a disruptive impact on the posterior ocular structures in chronic nature or severity of the disease and support the theory that MWA and MWoA may be not distinct disorders because they lead to the same results [39–41].

In conclusion, the peripapillary RNFL and choroid were significantly thinner in the migraine patients, with or without aura, than in the age- and sex-matched healthy control subjects. Our study suggests that migraine leads to a reduction in the peripapillary RNFL thickness and to thinning in choroidal structures in presence of the migraine as a result of the chronic nature and severity of disease. These findings can be explained by a chronic ischemic insult related to migraine pathogenic mechanisms and these findings are considered supportive of the relationship between glaucoma and migraine.

## Figures and Tables

**Table 1:** Participants' characteristics.

Variable	Control (*n* = 38)	Migraine type		*P *
		MWA (*n* = 36)	MWoA (*n* = 40)	
Age (years)	37.87 ± 11.08	38.8 ± 11.59	37.63 ± 15.72	0.939
Gender (male/female)	4 (10.5)/34 (89.5)	4 (11.1)/32 (88.9)	4 (10.0)/36 (90)	0.937
BCVA (log⁡MAR)	0.013 ± 0.034	0.013 ± 0.043	0.012 ± 0.035	0.999
IOP (mmHg Appl.)	14.4 ± 3.1	14.5 ± 2.7	15.1 ± 2.9	0.833
AL (mm)	23.34 ± 0.81	23.44 ± 0.85	23.20 ± 0.67	0.722
Duration of migraine (years)	—	10.8 ± 8.1	11.1 ± 7.9	0.899
Number of migraine attacks/month	—	3.1 ± 1.6	2.9 ± 1.6	0.693

Values are expressed as *n* (%), mean ± SD.

**Table 2:** Comparison of the peripapillary retinal nerve fiber layer (RNFL) thickness (*μ*m) of the migraine types and control groups.

Variable	Control (*n* = 38)	Migraine type		*P* ^†^	*P* ^‡^
		MWA (*n* = 36)	MWoA (*n* = 40)		
RNFL-G	102.18 ± 8.11	88.38 ± 18.01	84.8 ± 28.12	0.075	0.139
RNFL-T	76.53 ± 13.27	68.34 ± 18.43	71.6 ± 14.74	0.111	
RNFL-TS	137.84 ± 19.55	119.41 ± 30.28	114.2 ± 49.52	0.179	
RNFL-NS	113.84 ± 20.4	95.34 ± 24.83	92.4 ± 41.25	0.111	
RNFL-N	76.24 ± 11.34^a^	67.97 ± 15.33^b^	63.2 ± 16.5^b^	0.018	
RNFL-Nİ	116.84 ± 23.62^a^	98.1 ± 21.89^b^	91.6 ± 37.64^b^	0.003	
RNFL-Tİ	141.11 ± 23.49	120.86 ± 35.83	109.2 ± 46.74	0.100	

*P* ^†^: significance value after one-way analysis of variance (ANOVA). *P* ^‡^: significance value after one-way multivariate analysis of variance (MANOVA). Values are expressed as mean ± SD. Different superscripts (a, b) in a row indicate statistically significant difference among groups.

**Table 3:** Comparison of choroid thickness (*µ*m) of the migraine types and control groups.

Variable	Control (*n* = 38)	Migraine type		*P* ^†^	*P* ^‡^
		MWA (*n* = 36)	MWoA (*n* = 40)		
Subfoveal choroid thickness	339.13 ± 52.92^a^	278.37 ± 37.8^b^	283.5 ± 32.92^b^	<0.001	<0.001
Temporal choroid thickness	325.55 ± 53.93^a^	262.47 ± 35.13^b^	271.88 ± 32.94^b^	<0.001	
Nasal choroid thickness	317.13 ± 52.42^a^	255.03 ± 35.8^b^	261.13 ± 34.55^b^	<0.001	

*P* ^†^: significance value after one-way analysis of variance (ANOVA). *P* ^‡^: significance value after one-way multivariate analysis of variance (MANOVA). Values are expressed as mean ± SD. Different superscripts (a, b) in a row indicate statistically significant difference among groups.

**Table 4:** Comparison of the mean macular thickness (*μ*m) of the migraine types and control groups.

Variable	Control (*n* = 38)	Migraine type		*P* ^†^	*P* ^‡^
		MWA (*n* = 36)	MWoA (*n* = 40)		
Central macular thickness (1 mm)	262.61 ± 20.01	267.46 ± 21.29	260.2 ± 17.8	0.601	0.195
Temporal inner macula	333.78 ± 20.87	342.83 ± 14.52	342.0 ± 6.04	0.150	
Superior inner macula	349.97 ± 15.13	350.54 ± 14.89	349.8 ± 7.4	0.988	
Nasal inner macula	342.47 ± 18.1	338.92 ± 19.23	342.6 ± 15.4	0.752	
Inferior inner macula	342.5 ± 14.57	344.88 ± 13.17	339.4 ± 18.96	0.686	
Temporal outer macula	302.64 ± 22.45	315.08 ± 22.93	318.4 ± 16.18	0.069	
Superior outer macula	309.06 ± 12.29	307.42 ± 11.44	311.2 ± 4.02	0.759	
Nasal outer macula	307.44 ± 39.67	302.08 ± 33.32	310 ± 22.41	0.825	
Inferior outer macula	295.75 ± 11.76	300.33 ± 13.06	298.4 ± 9.32	0.360	

*P* ^†^: significance value after one-way analysis of variance (ANOVA). *P* ^‡^: significance value after one-way multivariate analysis of variance (MANOVA). Values are expressed as mean ± SD. Different superscripts in a row indicate statistically significant difference among groups.
